# Tracking the immunopathological response to *Pseudomonas aeruginosa* during respiratory infections

**DOI:** 10.1038/srep21465

**Published:** 2016-02-17

**Authors:** Cristina Cigana, Nicola Ivan Lorè, Camilla Riva, Ida De Fino, Lorenza Spagnuolo, Barbara Sipione, Giacomo Rossi, Alessandro Nonis, Giulio Cabrini, Alessandra Bragonzi

**Affiliations:** 1 Division of Immunology, Transplantation and Infectious Diseases, IRCCS San Raffaele Scientific Institute, Milano, Italy; 2 School of Biosciences and Veterinary Medicine, University of Camerino, Italy; 3 University Center for Statistics in the Biomedical Sciences (CUSSB), Vita-Salute San Raffaele University, Milan, Italy; 4 Department of Pathology and Diagnostics, University Hospital, Verona, Italy

## Abstract

Repeated cycles of infections, caused mainly by *Pseudomonas aeruginosa,* combined with a robust host immune response and tissue injury, determine the course and outcome of cystic fibrosis (CF) lung disease. As the disease progresses, *P. aeruginosa* adapts to the host modifying dramatically its phenotype; however, it remains unclear whether and how bacterial adaptive variants and their persistence influence the pathogenesis and disease development. Using *in vitro* and murine models of infection, we showed that *P. aeruginosa* CF-adaptive variants shaped the innate immune response favoring their persistence. Next, we refined a murine model of chronic pneumonia extending *P. aeruginosa* infection up to three months. In this model, including CFTR-deficient mice, we unveil that the *P. aeruginosa* persistence lead to CF hallmarks of airway remodelling and fibrosis, including epithelial hyperplasia and structure degeneration, goblet cell metaplasia, collagen deposition, elastin degradation and several additional markers of tissue damage. This murine model of *P. aeruginosa* chronic infection, reproducing CF lung pathology, will be instrumental to identify novel molecular targets and test newly tailored molecules inhibiting chronic inflammation and tissue damage processes in pre-clinical studies.

The genetic defect underlying cystic fibrosis (CF) disrupts lung function by obstruction with thick, sticky secretions which predispose to infections, as those by *Pseudomonas aeruginosa*^1^. Early in life CF lungs are characterized by intermittent airway bacterial infections and excessive neutrophil-dominated inflammation^2^. Later, chronic infection with *P. aeruginosa* fuels a sustained, exuberant inflammatory response that progressively destroys the lungs and ultimately results in respiratory failure. Structural changes in CF lungs include bronchiectasis, airway mucus plugging, microabscesses, peribronchial inflammation, fibrosis -associated with increased levels of metalloproteinases (MMPs)^3^,^4^,^5^ and glycosaminoglycans (GAG)^6^,^7^,^8^- and vascular changes, all indicating extensive remodeling that contributes to the lung function decline^9^,^10^,^11^.

Long-term chronic infection is carried on by *P. aeruginosa* variants characterized by adaptive traits^12^,^13^. Indeed, the genetic and phenotypic properties of persisting bacterial cells in CF airways differ greatly from those that initiated the infections^12^. The findings that chronic infection in CF patients progresses with bacteria lacking invasive virulence functions open the questions whether: i) *P. aeruginosa* CF-adapted variants live life-long in the lung avoiding host immune response and/or ii) their persistence triggers pathways relevant for tissue remodelling, that finally lead to lung function decline. These issues are difficult to approach in humans due to confounding factors, such as the diverse polymicrobial community, unknown environmental factors and host genetic variability.

Several animal models of *P. aeruginosa* infection as well as CF mouse models have been generated^14^. Most of them are focused on modelling the acute phases of the *P. aeruginosa* infection, while others use immobilizing agent such as agar, agarose, or seaweed alginate to monitor certain aspects of the chronic infection in mice^15^. However, at the time of this writing no experimental studies have managed to model the advanced-stage of lung pathology that strictly mimics human CF infection^14^. Pulmonary damage, remodeling and fibrosis are more difficult to be reproduced. While these pathological traits of human disease can be induced by causative agents (e.g. bleomycin), they have not been reproduced following bacterial pneumonia in mice. As a consequence, the cascade of events mediated by *P. aeruginosa* persistence in the pathogenesis of chronic airways infection has been difficult to address.

Using models of infection, we demonstrated that *P. aeruginosa* CF-adapted isolates shape innate immunity to favor their persistence. Remarkably, we succeeded in reproducing the advanced stage of chronic infection lasting for three months with stable bacterial load and traits of the CF human airway disease in mice, including airway remodeling and damage.

## Results

### *P. aeruginosa* CF-adapted variants shape the host immune response during the progression of infection

**Figure 1:**
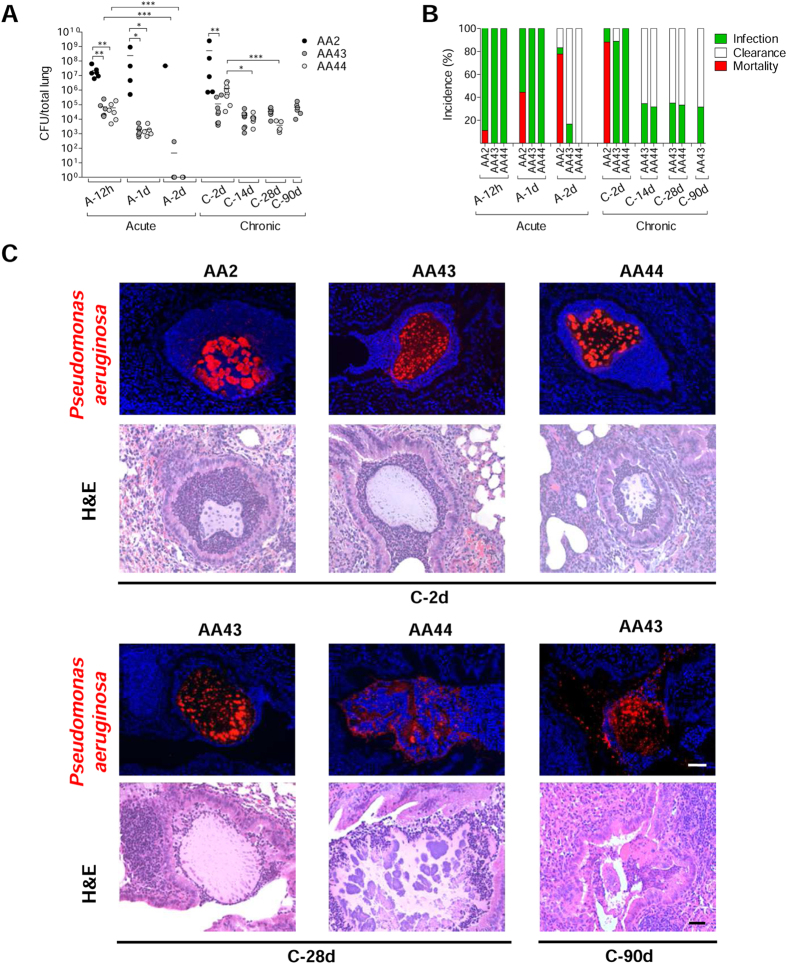
Virulence of *P. aeruginosa* isolates and bacterial localization in murine models of airways infection. Two groups of minimum five C57Bl/6NCrlBR mice were infected with 5 × 10^6^ CFU/lung of planktonic bacteria for the acute infection (data modified from Fig. 5P included in Lorè NI *et al.* PLoS One 2012;7(4):e35648.) and with 1 to 2 × 10^6^ CFU/lung of isolates embedded in agar beads for the chronic infection and analyzed during a time course post-infection (12 hours, 1 and 2 days of acute infection and 2, 14, 28 and 90 days of chronic lung infection). (**A**) CFUs were evaluated in total lung. Dots represent CFUs in individual mice and horizontal lines represent median values. The data are pooled from at least two independent experiments (n = 1–9). Statistical significance is indicated: *p < 0.05, **p < 0.01, ***p < 0.001. (**B**) The incidences of mortality induced by bacteremia (red), clearance (white) and airway infection (green) were determined. The data are pooled from at least two independent experiments (n = 8–24). (**C**) Bacterial and agar-beads localization in the lung were evaluated on challenged mice by immunofluorescence (with specific antibody against *P. aeruginosa*, stained in red, and with 4,6-Diamidino-2-phenylindole dihydrochloride, stained in blue) and H&E staining. Scale bar: 25 μm.

To track the host response with *P. aeruginosa* variants, acute (planktonic bacteria) and chronic (agar-bead embedded bacteria) infections were established in C57BL/6NCrlBR mice with CF-adapted AA43 and AA44 in comparison with AA2 clonal isolate. In particular, AA43 and AA44 were, respectively, mucoid and non-mucoid variants with adaptive phenotypes, including absence of swimming motility, low twitching motility and production of protease, while AA2 expressed a wide series of virulence factors including swimming motility, twitching motility and protease secretion (Fig. S1)^16^,^17^. AA2 either disseminated systemically and induced death or was cleared by the host in both acute^17^ and chronic murine models of lung infection confirming previous data^16^ (Fig. 1A,B). Planktonic bacteria of AA43 and AA44 CF-adapted variants were cleared after acute infection^17^, while bacteria embedded in agar-beads retained their capacity to persist in murine lungs. Here, chronic colonization was extended up to three months with bacterial loads stabilizing at ≈10^4^ colony forming units (CFU)/lung. Thus, the susceptibility to *P. aeruginosa* chronic infection is isolate-dependent in our murine model.

**Figure 2:**
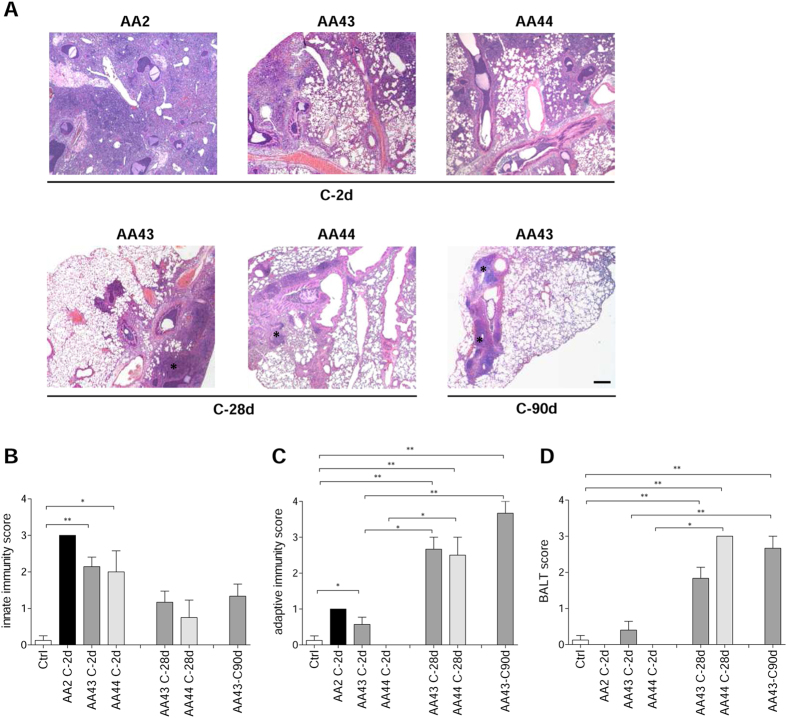
Lung histology and histopathological score of immune cells recruitment in the murine model of *P. aeruginosa* chronic airways infection. C57Bl/6NCrlBR mice were infected with 1 to 2 × 10^6^ CFU/lung of isolates embedded in agar beads for the chronic infection and analyzed during a time course post-infection (2, 28 and 90 days). (**A**) Lung histopathology was performed on challenged mice by H&E staining. Scale bars: 200 μm. BALT-like structures are indicated by asterisks. Innate (**B**) and adaptive (**C**) immune cells infiltration and BALT activation (**D**) were scored in tissue section of murine lungs stained with H&E. The data are pooled from at least two independent experiments (n = 2–7). Values represent the mean ± standard error of the mean (SEM). Statistical significance is indicated: *p < 0.05, **p < 0.01.

**Figure 3:**
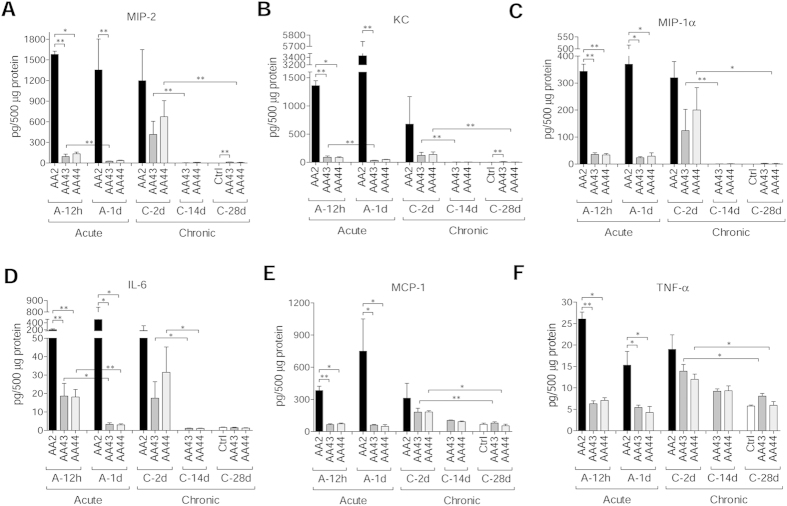
Cytokines/chemokines profiles in murine models after *P. aeruginosa* infection. C57Bl/6NCrlBR mice were infected with 5 × 10^6^ CFU/lung of planktonic bacteria for the acute infection or 1 to 2 × 10^6^ CFU/lung of strains embedded in agar beads for the chronic infection and analyzed during a time course post-infection (12 hours and 1 day of acute infection and 2, 14 and 28 days of chronic lung infection). Cytokines and chemokines, including MIP-2 (**A**), KC (**B**), MIP-1α (**C**), IL-6 (**D**), MCP-1 (**E**) and TNF-α (**F**), were measured by Bioplex in lung homogenates. The data are pooled from at least two independent experiments (n = 3–6). Values represent the mean ± SEM. Statistical significance is indicated: *P < 0.05, **P < 0.01.

Immunofluorescent staining for *P. aeruginosa* and Haematoxylin and Eosin (H&E) on day 2 localized the infection to bronchial lumens in the agar-beads (Fig. 1C). In the initial phases, infection with the AA43 and AA44 CF-adapted variants yielded lower recruitment of innate immune cells, including neutrophil and macrophage (Fig. 2A,B), and less MIP-2, KC, MIP-1α, IL-6, MCP-1 and TNF-α cytokines/chemokines production than AA2 (Fig. 3A–F). After 28 and 90 days, the persistent AA43 and AA44 CF-adapted variants, irrespective of mucoid phenotype, were localized as macrocolonies in the beads remaining in the bronchial lumen and in biofilm-like structures (Fig. 1C). At this stage, cytokines/chemokines decreased further, but were still detectable in mice retaining infection with AA43 and AA44 isolates (Fig. 3A–F). The persistence of CF-adapted variants promoted an adaptive immune response characterized also by the formation of bronchus-associated lymphoid tissue (BALT)-like structures (Fig. 2A,C,D). Overall, *P. aeruginosa* variants with CF-adapted phenotype determine a weakening of the innate immune recognition and a shift in the immune response during the course of airway diseases.

### Differential cell host response to *P. aeruginosa* phenotypic variants

**Figure 4:**
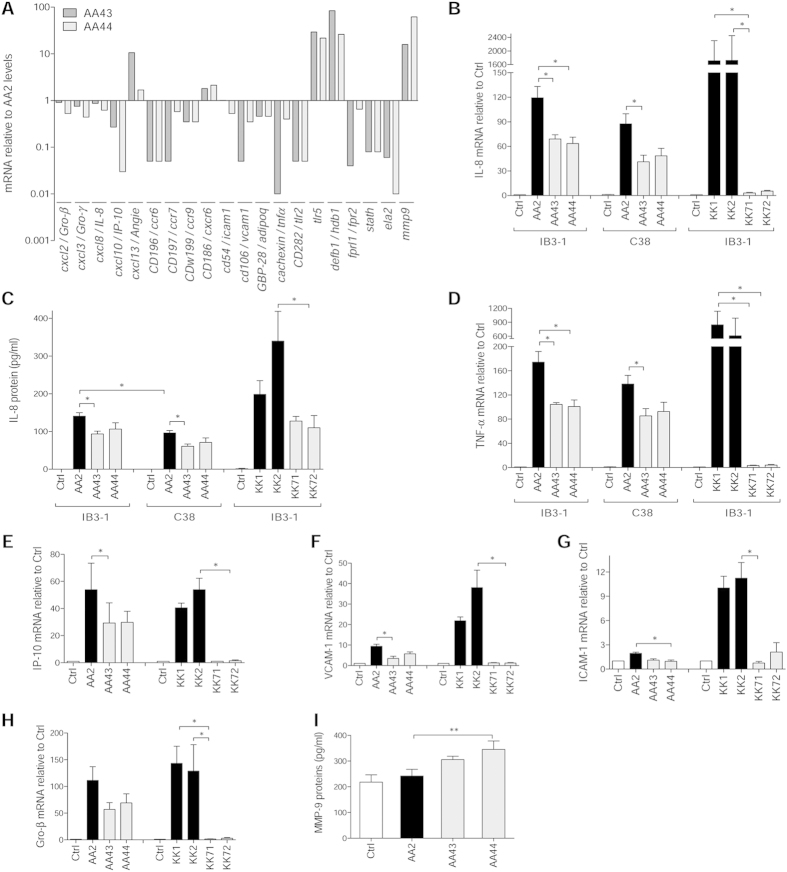
Expression/release of markers of inflammation and tissue damage in cell lines after infection with *P. aeruginosa* phenotypic variants. Bronchial epithelial CF cells IB3-1 were infected for 4 hours with *P. aeruginosa* AA2-AA43-AA44 isolates, RNA extracted and retrotranscribed, and macroarray conducted. (**A**) Genes expression is expressed after normalization on expression induced by AA2. Validation of gene expression was performed by real time PCR in IB3-1 (**B**,**D**–**H**) and isogenic non-CF cells C38 (**B**,**D**) after infection with AA2, AA43, AA44, KK1, KK2, KK71 and KK72. (**C**) Validation of IL-8 protein release was performed by ELISA in culture medium of IB3-1 and C38 after infection with isolates mentioned above. (**I**) Macrophagic-like cells THP-1 were infected for with *P. aeruginosa* AA2, AA43 and AA44 isolates (MOI 1), and MMP-9 release was measured in the culture supernatants by ELISA. Values represent the mean ± SEM. The data are pooled from at least three independent experiments. Statistical significance is indicated: *p < 0.05, **p < 0.01.

Next, we conducted *in vitro* analysis to exclude the confounding variables (e.g. different bacterial load) during the course of the infection that cannot be controlled in mouse models. Thus, RNA macroarray analysis^18^ of human IB3-1 cells infected with AA2 and CF-adapted AA43 and AA44 variants was performed to investigate the host response mediated by bronchial epithelial cell models widely investigated in CF inflammation. We identified a total of 20 genes whose transcription was changed more than 2-fold in cells infected with different *P. aeruginosa* variants but with equal bacterial load (Fig. 4A, Table S1). Only five genes were up-regulated after infection with the AA43 and AA44 CF-adapted variants in respect to AA2 infection, while the majority (15 out of 20) were down-regulated, including several chemokines and cytokines and their receptors (TNF-α, Gro-β and γ, IP-10, IL-8, FPR2, CCR6, CCR7, CCR9), and adhesion molecules involved in the process of leukocytes extravasation and recruitment (VCAM-1 and ICAM-1). In addition, although AA2 isolate induced higher expression of elastase 2, liable for collagen-IV and elastin proteolysis, AA43 and AA44 induced much higher expression of the matrix metalloprotease 9 (MMP-9), suggesting a contribution of *P. aeruginosa* CF-adapted variants in matrix degradation. Validation of results by real time PCR and/or ELISA for selected targets (IL-8, TNF-α, IP-10, VCAM-1, ICAM-1, Gro-β, MMP-9) has been carried out in different cell types (IB3-1, C38 and THP-1) (Fig. 4B–I).

Next, we extended our investigation to additional *P. aeruginosa* CF-adaptive variants (Fig. 4B–H). Selection was based on previous characterization: KK71 and KK72 showed phenotypic traits of CF-adaptation absent in KK1 and KK2 clonal variants (Fig. S1)^16^,^17^. Results confirmed the lower pro-inflammatory potential of CF-adapted KK71 and KK72 in comparison with KK1 and KK2 variants. Overall, these results unravel that *P. aeruginosa* CF-adapted variants rewrite the host response in CF bronchial epithelial cell lines and in particular attenuate the expression of a set of genes involved in inflammation, confirming our hypothesis with additional *P. aeruginosa* variants.

### *P. aeruginosa* persistence promotes airway tissue damage in mice that mirrors human CF pathology

**Figure 5:**
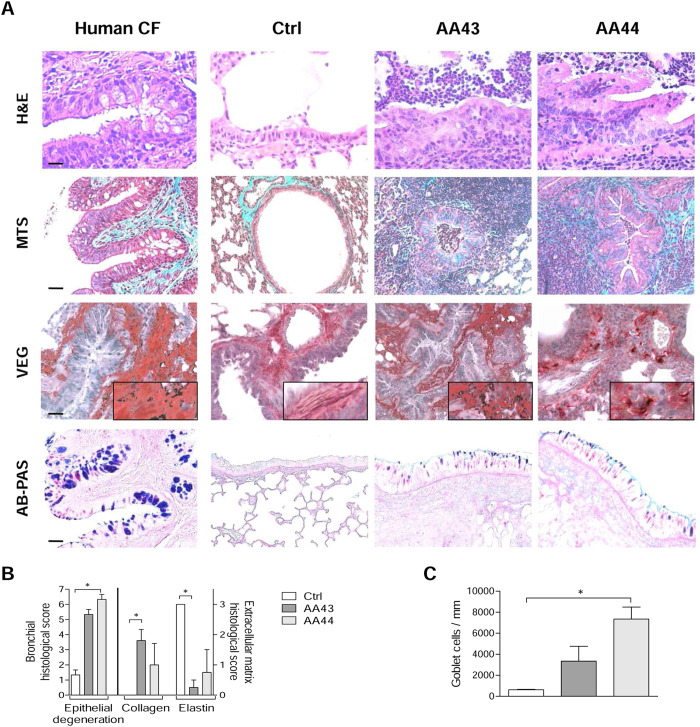
Lung histology and histopathological score of tissue damage in murine lung after infection with *P. aeruginosa*. C57Bl/6NCrlBR mice were infected with 1 to 2 × 10^6^ CFU/lung of isolates embedded in agar beads and analyzed 28 days post-infection. Sections of airways from transplanted CF patients and from C57Bl/6NCrlBR infected with CF-adapted isolates AA43 and AA44 and sterile beads were stained with H&E, MTS for collagen, VEG for elastic fibers and AB/PAS for mucopolysaccarides, according to the standard procedures (**A**). Scale bars: 12.5 μm for H&E, 25 μm for MTS, VEG and AB-PAS. Scorings of bronchial epithelial degeneration, collagen deposition and elastin degradation (**B**) were performed on slices stained with H&E, MTS and VEG, respectively. Goblet cells numbers were evaluated on slices stained with AB-PAS (**C**). The data are pooled from at least two independent experiments (n = 2–6). Values represent the mean ± SEM. Statistical significance is indicated: *p < 0.05.

**Figure 6:**
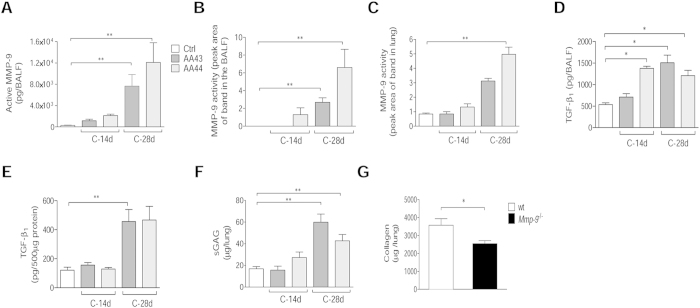
Tissue damage markers, including MMP-9, in murine models after *P. aeruginosa* infection. C57Bl/6NCrlBR mice were infected with 1 to 2 × 10^6^ CFU/lung of CF-adapted isolates embedded in agar beads for 14 and 28 days. Levels of MMP-9 protein (**A**) in BALF by ELISA, MMP-9 activity (**B**) in BALF and (**C**) lung homogenate by zymography, TGF-β_1_ (**D**) in BALF and (**E**) lung homogenate by Bioplex and sGAG (**F**) in lung homogenate by a dye-binding colorimetric assay were measured after 14 and 28 days of chronic lung infection. Values represent the mean ± SEM. The data are pooled from at least two independent experiments (n = 3–12). **G**) B6.FVB(Cg)-Mmp9tm1Tvu/J and congenic mice were infected with 2 × 10^6^ CFU/lung of AA43 strain embedded in agar beads. Collagen levels were evaluated by a dye-binding assay in lung homogenate after 28 days of chronic lung infection with the *P. aeruginosa* CF-adapted isolate AA43. Values are represented as mean ± SEM. The data derive from one experiment (n = 6–7). Statistical significance is indicated: *p < 0.05, **p < 0.01.

Next, we investigated whether and to what extent chronic persistence by *P. aeruginosa* CF-adapted variants recapitulates human CF lung disease (Fig. 5). Mice, retaining infection with AA43 and AA44 CF-adapted variants for one month, displayed hallmarks of CF chronic lung pathology: intraluminal and peribronchial inflammation, epithelial hyperplasia and structure degeneration, goblet cell metaplasia, collagen deposition and elastin degradation (Fig. 5A–C). Biochemical markers of tissue damage in CF disease such as MMP-9 activity and protein, TGF-β_1_ protein and sulphated GAG (sGAG) increased progressively during long-term infection with AA43 and AA44 CF-adapted variants (Fig. 6A–F). So for the first time, the above described mouse model of long-term airway chronic infection established with *P. aeruginosa* CF-adapted variants reproduced several traits of human CF lung pathology.

Taking into consideration the emerging relevance of MMPs to pulmonary remodelling and impaired lung function in CF patients^6^,^19^,^20^, and MMP-9 activation in our chronic infection model and cell culture, we investigated host response to chronic infection by AA43 CF-adapted isolate in MMP-9 deficient mice. Lower collagen levels were found in *Mmp-9*^−/−^ mice compared to the isogenic counterpart (Fig. 6G) while no differences in terms of bacterial load, inflammatory cells recruitment, sGAG and TGF-β_1_ after one month were recorded (Fig. S2). These findings suggest a role of MMP-9 in the process of collagen deposition during *P. aeruginosa* chronic persistence.

### Contribution of CFTR-deficiency to the pathogenesis of *P. aeruginosa* infection

**Figure 7:**
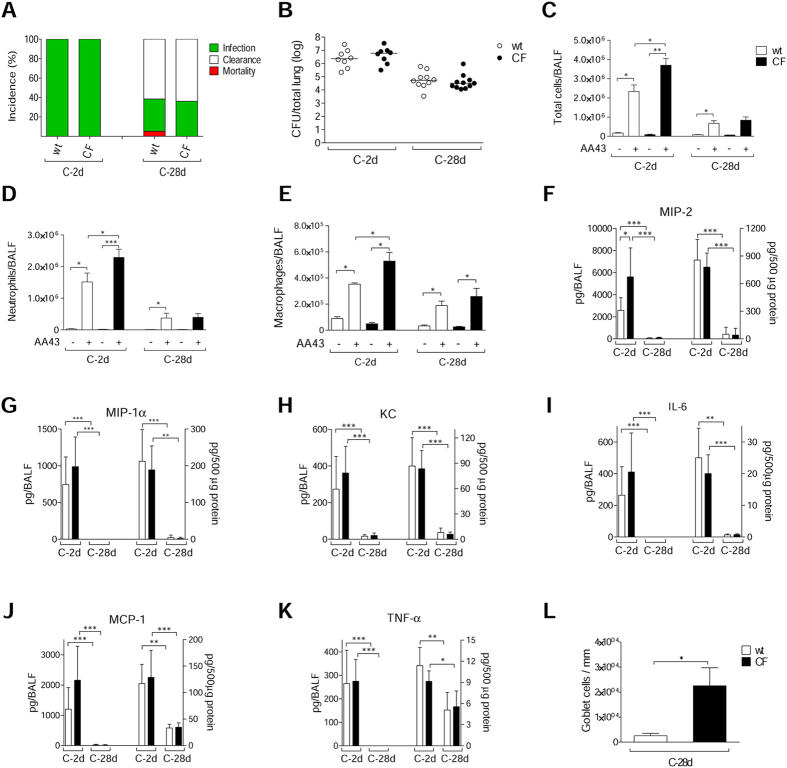
Bacterial virulence and lung inflammatory response in CF and congenic wt mice after *P. aeruginosa* chronic lung infection. Gut-corrected CFTR-deficient C57Bl/6 Cftr^tm1UNC^TgN(FABPCFTR)#Jaw and congenic wt mice were infected with 2 × 10^6^ CFU/lung of *P. aeruginosa* AA43 strain embedded in agar beads and analyzed after 2 and 28 Days. **A**) The incidences of mortality induced by bacteremia (red), clearance (white) and airway infection (green) were determined. The data are pooled from two independent experiments (n = 8–28). **B**) CFU were evaluated in total lung. Dots represent individual mice measurements and horizontal lines represent the median values reported in a log scale (n = 8–11). The number of total cells (**C**) and in particular of neutrophils (**D**) and macrophages (**E**) in the airways were analyzed in BALF of mice. Cytokines/chemokines, including MIP-2 (**F**), MIP-1α (**G**), KC (**H**), IL-6 (**I**), MCP-1 (**J**) and TNF-α (**K**), were measured by Bioplex in murine BALF (left side of the graph) and lung homogenates (right side of the graph). Sections of murine lungs infected with AA43 for 28 days were stained with AB/PAS for mucopolysaccharides, according to the standard procedure. Goblet cells numbers were evaluated on slices stained with AB-PAS (**L**). Values represent the mean ± SEM. The data are pooled from at least two independent experiments (n = 3–11). Statistical significance is indicated: *p < 0.05, **p < 0.01, ***p < 0.001.

To determine whether the CFTR-deficiency could exacerbate the inflammatory response or tissue damage after chronic *P. aeruginosa* infection, we infected gut-corrected CFTR-deficient [C57Bl/6 Cftr^tm1UNC^TgN(FABPCFTR)#Jaw] and their congenic wt mice with the AA43 CF-adapted variant. The percentage of *P. aeruginosa* infection and bacterial load were similar between CF and wt mice, revealing the capacity of *P. aeruginosa* AA43 CF-adapted isolate to persist in murine lungs regardless of the CF genetic background (Fig. 7A,B). However, at the early time point the inflammatory response following *P. aeruginosa* infection in the bronchoalveolar lavage fluid (BALF) was higher in CF mice, in terms of total cells, neutrophils, macrophages and MIP-2 levels, compared to wt mice (Fig. 7C–F). KC, MIP-1α, IL-6, MCP-1 and TNF-α in BALF were similar between CF and isogenic wt mice (Fig. 7G–K). The chronic BALF response was similar in CF and wt animals. In the lung homogenate, the levels of all of the above-mentioned chemokines/cytokines were similar between CF and wt mice at both the early and late chronic time points. At an advanced stage of chronic infection, CF and wt mice differed in terms of goblet cell metaplasia. Histology and pathological scores revealed a higher number of goblet cells in the lung tissue of CF mice compared to wt, indicating greater mucus production (Figs 7L and S3A). Other markers of tissue damage, such as collagen deposition, elastin degradation, MMP-9 activity, sGAG and TGF-β_1_, were similarly increased in CF and wt mice (Fig. S3B–F). Furthermore, no differences were detected in recruitment of innate and adaptive immune cells, and BALT formation (data not shown). Overall, these data indicate that, in mice, alteration of the immune response and subsequent tissue remodeling during *P. aeruginosa* chronic infection are mostly independent of the presence of functional CFTR.

## Discussion

Current debates on CF pathogenesis recognize that multifactorial variables contribute to the outcome of lung disease; these include host factors and the bacterial pathogenic potential^21^,^22^. Dissection of these factors and their causal associations in humans are complicated by confounding variables that cannot be controlled individually. Here, using well established *in vitro* and *in vivo* models of infection we demonstrated that *P. aeruginosa* CF-adapted variants shape the innate immune response to favor their persistence. However, the previous lack of a suitable animal model of long-term chronic infection has limited understanding of the consequence of *P. aeruginosa* persistence on host response. In this study, we refined the agar-beads mouse model approaching the advanced stage of chronic pneumonia. This model was instrumental in demonstrating the contribution of *P. aeruginosa* long-term persistence in activating relevant pathways for tissue remodeling and damage. Surprisingly, *P. aeruginosa* persistence had a greater effect on inflammation and damage profile rather than the *Cftr* mutation itself. These findings have real importance to understanding CF airways disease pathogenesis and progression.

For this study, we used *P. aeruginosa* isolates sampled at the onset of infection and after years of chronic colonization; they were selected for their diversity determined in previous characterization^16^,^17^,^23^. The *P. aeruginosa* CF-adapted isolates were genetically characterized by genome rearrangements, mutations, and variations in pathogenic islands, and phenotypically by altered motility, mucoidy, and changes in virulence factors. Thus, the selected *P. aeruginosa* isolates display features commonly associated with bacterial adaptation to CF lung^12^,^13^,^16^. When we investigated *P. aeruginosa*/host interaction in mice, the *P. aeruginosa* isolate equipped with virulence factors either disseminated systemically resulting in death or was cleared by a strong host inflammatory response, while CF-adapted variants diminished the innate immune response to allow long-term persistence. Indeed, by measuring cytokines/chemokines at acute/early phases of infection, we found lower pro-inflammatory response induced by *P. aeruginosa* adapted variants. One factor of major interest was that the host response of *P. aeruginosa* adapted variants did not appear to be dependent only on a particular bacterial phenotype or clonal variants. Different *P. aeruginosa* patho-adaptive variants (e.g. mucoid and a non-mucoid) or belonging to different patients, showed a similar capacity to shape the host immune response.

Although mice deficient for *Cftr*^24^, or overexpressing the β-ENaC channel^25^, have been available for many years, these models do not completely mimic the advanced human pathology^26^,^27^,^28^,^29^. Infection of CF mice with *P. aeruginosa* has been performed and resulted in a heightened inflammatory response^30^, but high mortality or resolution of the infection have typically limited pathogen persistence for long time. Lung pathology in β-ENaC mice reflects undeniably several hallmarks of CF airways disease, including mucus hyperproduction, goblet cells metaplasia, neutrophilic inflammation and poor bacterial clearance^31^,^32^. However, they do not reproduce spontaneously the advanced stage of CF lung disease, including chronic *P. aeruginosa* infection, the development of the adaptive immune response and a considerable tissue damage (e.g. elastin degradation and collagen deposition). Models of chronic infections have been described but, at the time of writing, no experimental studies have managed to achieve the progressive advanced-stage of lung pathology induced by chronic bacterial colonization that strictly mimics human CF infection (e.g., chronic Pseudomonal endobronchitis). Although models of long-term chronic infection have been described and are undeniably useful to study several features of CF lung disease including *P. aeruginosa* evolution or chronic inflammation, they show some weaknesses, such as the absence of a stable bacterial load over the course of the infection or the use of mechanical devices as catheters that render, the model not-physiological^33^,^34^. The previous lack of an accessible CF animal model has limited not only a satisfactory evaluation of the host adaptive immune response, but also the understanding of tissue damage and remodeling processes observed during chronic pneumonia, as described in CF disease^15^,^35^. We successfully reproduced the long-term chronic infection with stable bacterial titers up to three months and the presence of bacteria in biofilm-like structures. In this model, a long-term consequence of *P. aeruginosa* chronic infection is the formation of BALT-like structures. Moreover, the degeneration of the epithelial structure, collagen deposition, elastin degradation, increased factors associated with matrix remodeling (MMP-9 activity, sGAG and TGF-β_1_) and goblet cell metaplasia were also observed in murine airways. This immunopathology is notably similar to the clinical descriptions of human CF lung disease, mirroring chronic Pseudomonal endobronchitis of the CF population.

In our CF mouse model, we found that the CFTR deficiency was associated with a higher inflammatory response to *P. aeruginosa* at the early phases of infection which is in agreement with previous observations^15^. When we extended the infection to the advanced stage, tissue remodeling and damage were partially independent of the *Cftr* murine background. Interestingly, our data showed that pathological traits developed similarly in both wt and CF mice, with further increase of goblet cell metaplasia in the CF host. In this context, the pro-inflammatory environment of the lung can impair and decrease CFTR function as demonstrated in chronic obstructive pulmonary disease (COPD)^36^,^37^. Furthermore, recent observations indicate that TGF-β_1_ and hypoxic environments down-regulate the expression and activity of CFTR^38^,^39^. Thus, it could be speculated that chronic inflammatory profile and high levels of TGF-β_1_ after long-term *P. aeruginosa* infection in wt mice may undermine CFTR function and dampen the differences between wt and CF mice. Further studies are required to strengthen these hypotheses. Most important, the findings that CFTR function could be impaired by *P. aeruginosa* infection, its virulence factors and the associated-chronic inflammation^40^ may further explain the similar pathological events developed independently of the CFTR deficiency in our murine model. Overall our data strongly suggest that *P. aeruginosa* persistence is a key driver of the pathogenesis of CF chronic lung disease. Relevance of these studies may be extended to other chronic endobronchitis including advanced COPD, where some hallmarks observed in CF, such as high inflammation, tissue remodeling and *P. aeruginosa* persistence, have been described^41^.

New therapeutic approaches to preserve airway structure and pulmonary function are a clinical need. Taking into consideration the emerging relevance of MMPs to pulmonary remodelling in CF patients^6^,^19^,^20^ and MMP-9 link to *P. aeruginosa* persistence, observed in this work, we further investigated its role in the pathophysiology of the chronic disease. Results obtained in *Mmp*-9^−/−^ mice demonstrated that this metalloprotease contributes to collagen deposition during *P. aeruginosa* chronic infection, while it does not exert any harmful effect on host defense. In agreement with other recent studies^42^, our results may support the exploitation of this target in CF.

In conclusion, our study demonstrates that *P. aeruginosa* CF-adaptive variants shape the host response favoring their persistence within the lung. Furthermore, the *P. aeruginosa* persistence contributes to modulate pathways relevant for airway structural changes, reproducing human pathology in mice. Notably, the mouse model, refined in this study, is an invaluable tool for studying the pathogenesis and progression of infection in CF and other chronic airway diseases (such as advanced COPD) and may be an ideal platform for testing novel therapies. In addition, our findings emphasize that the development of new therapeutic interventions (e.g., drug/pharmacological) for CF should target tissue remodeling and damage and account for immune changes that manifest in the setting of chronic infection.

## Methods

### Ethics Statement

Animal studies were conducted according to protocols adhering strictly to the Italian Ministry of Health guidelines for the use and care of experimental animals (IACUC protocols #402 and 502) and approved by the San Raffaele Scientific Institute (Milan, Italy) Institutional Animal Care and Use Committee (IACUC).

The use of human bronchi, obtained from patients undergoing lung transplant, was approved by the Ethical Committee of the Gaslini Institute (Genova, Italy), in accordance with the guidelines of the Italian Ministry of Health. Each patient provided written informed consent to the study using a form that was also approved by the Ethical Committee (approval #13).

Research with the longitudinal bacterial isolates from CF individuals (AA2, AA43, AA44, KK1, KK2, KK71 and KK72), used for *in vitro* and animal experiments, has been approved by the Ethics Commission of Hannover Medical School, Germany. The patients and parents gave informed consent before the sample collection. Approval for storing of biological materials was obtained by the Ethics Commission of Hannover Medical School, Germany.

### Bacterial strains

Sequential *P. aeruginosa* isolates from CF patients were chosen from a strain collection, previously characterized for genotypic and phenotypic traits, and virulence^16^,^17^,^23^. AA2, KK1 and KK2, expressing virulence factors, were isolated at the onset of chronic infection, while AA43, AA44, KK71 and KK72, showing different adaptive phenotypes, including mucoidy for AA43, were collected after 7 to 13 years after colonization.

### Mouse strains

C57Bl/6 (C57Bl/6NCrlBR, Charles River), MMP-9^−/−^ (B6.FVB(Cg)-Mmp9tm1Tvu/J, Jackson Laboratory) and congenic mice (C57Bl/6J, Jackson Laboratory), 8 to 10 weeks old, and CF [gut-corrected CFTR-deficient C57Bl/6 Cftr^tm1UNC^TgN(FABPCFTR)#Jaw] and congenic wild-type (wt) mice (originally obtained from Case Western Reserve University and maintained at San Raffaele Scientific Institute, Milan, Italy)^43^, 11 to 16 weeks old, were maintained in specific pathogen-free conditions.

### Mouse model of acute and chronic *P. aeruginosa* infection

Mice were injected intratracheally with 5 × 10^6^ CFU of planktonic *P. aeruginosa* or with 1–2 × 10^6^ CFU, embedded in agar beads, following established procedures^16^,^17^,^44^. The bacterial load was previously set-up as the minimum inoculum to establish chronic infection^45^. BALF and lung were recovered and processed, as previously described^44^.

### Histological examination

Sections of murine and human lungs for histological analysis were collected and stained by Haematoxylin and Eosin (H&E), Acid Schiff (AB-PAS), Masson’s trichrome (MTS), Verhoeff’s elastic (VEG) and immunofluorescence (IF) stainings and were examined blindly and scored by a pathologist, as detailed in Supplementary Information.

### Cell lines

IB3-1 cells, an adeno-associated virus-transformed human bronchial epithelial cell line derived from a CF patient (ΔF508/W1282X), and C38 cells, the cell line which expresses a plasmid encoding functional CFTR, and non-adherent human myelomonocytic THP1 cells were obtained from LGC Promochem. IB3-1 cells were grown as previously described^17^. *In vitro* infection was performed as described in Supplementary Information.

### Macroarray analysis

Macroarray analysis was performed by using TaqMan Low Density Array (TLDA) platform (Applied Biosystems, Foster City, CA), as detailed in Supplementary Information.

### Evaluation of Cytokines/chemokines and markers associated to tissue damage

Cytokines/chemokines and growth factors, MMP-9 active levels, sGAG and collagen, and MMP-9 activity were respectively measured by Bioplex, ELISA, colorimetric dye-binding assay and zymography, according to the manufacturer’s instructions as detailed in Supplementary Information.

### Statistics

Data analysis was performed using a nonparametric two-taled Mann-Whitney U test for single comparison when comparing data induced by a specific strain at two time points during acute infection (e.g. cytokines/chemokines levels) or comparison of data from wt and CF mice at each time-point. To compare data induced by CF-adapted variants to AA2 or Ctrl data at one time-point and data induced by a specific strain across multiple time-points a nonparametric Kruskal-Wallis test was used followed by post-hoc Dunn test to correct for multiple comparisons. Mortality and percentage of infection were compared using Fisher exact test. *P* < 0.05 was considered significant.

## Additional Information

**How to cite this article**: Cigana, C. *et al.* Tracking the immunopathological response to *Pseudomonas aeruginosa* during respiratory infections. *Sci. Rep.* **6**, 21465; doi: 10.1038/srep21465 (2016).

## Supplementary Material

Supplementary Information
