# Supplementary material for: Tracking the immunopathological response to Pseudomonas aeruginosa during respiratory infections
Source: Sci Rep. 2016 Feb 17;6:21465. doi: 10.1038/srep21465 (PMC4756310; doi:10.1038/srep21465)
Supplement: Supplementary Information [file srep21465-s1.pdf]

Tracking the immunopathological response to *Pseudomonas aeruginosa* during respiratory infections

Cristina Cigana, Nicola Ivan Lorè, Camilla Riva, Ida De Fino, Lorenza Spagnuolo, Barbara Sipione, Giacomo Rossi, Alessandro Nonis, Giulio Cabrini, Alessandra Bragonzi

Supplementary Information

SUPPLEMENTARY FIGURES AND LEGENDS

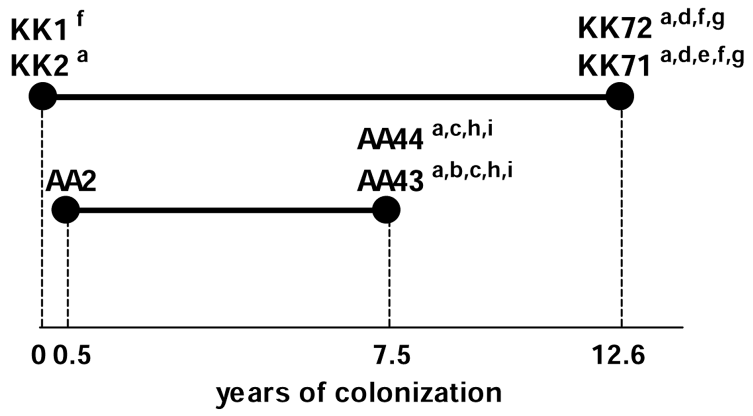

Fig. S1. Genotypic and phenotypic characteristic of *P. aeruginosa* sequential isolates from CF patients. Sequential *P. aeruginosa* isolates were recovered at the onset of chronic colonization (AA2, KK1, KK2) or several years after acquisition and before patient's death (AA43, AA44, KK71, KK72). Clonality of strains was assessed by Pulsed Field Gel Electrophoresis and was previously reported [1]. Multiple phenotypic traits changed during genetic adaptation to the CF lung [2] and included: (a) motility defect, (b) mucoid phenotype, (c) protease reduction, (d) siderophore reduction, (e) hemolysis reduction, (f) *LasR* mutant phenotype, (g) growth rate reduction. In addition, lipopolysaccharide (LPS) lipid A (h) and peptidoglycan (PGN) muropeptides (i) were analyzed exclusively in the AA isolates showing specific structural modifications temporally associated with CF lung infection as previously described [3].

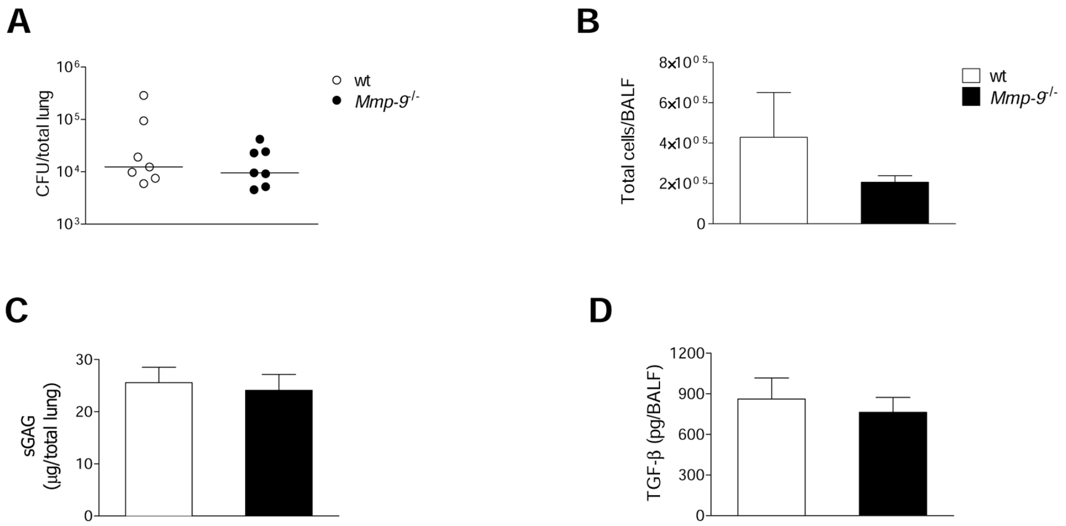

Fig. S2. Bacterial load, leukocytes recruitment and tissue damage in lungs of *Mmp-9*$^{-/-}$ and congenic wt mice after *P. aeruginosa* long-term chronic infection. B6.FVB(Cg)-Mmp9tm1Tvu/J and congenic mice were infected with $2x10^6$ CFU/lung of AA43 strain embedded in agar beads. **A**) CFU in total lung, **B**) total cells recruitment in BALF, **C**) sGAG in lung homogenate by a colorimetric assay and **D**) TGF-β_1 by Bioplex were evaluated after 28 days of chronic lung infection with the *P. aeruginosa* CF-adapted isolate AA43. Dots represent CFUs in individual mice and horizontal lines represent median values. Total leukocytes, sGAG and TGF-β_1 values are represented as mean ± SEM. The data derive from one experiment (n=6-7).

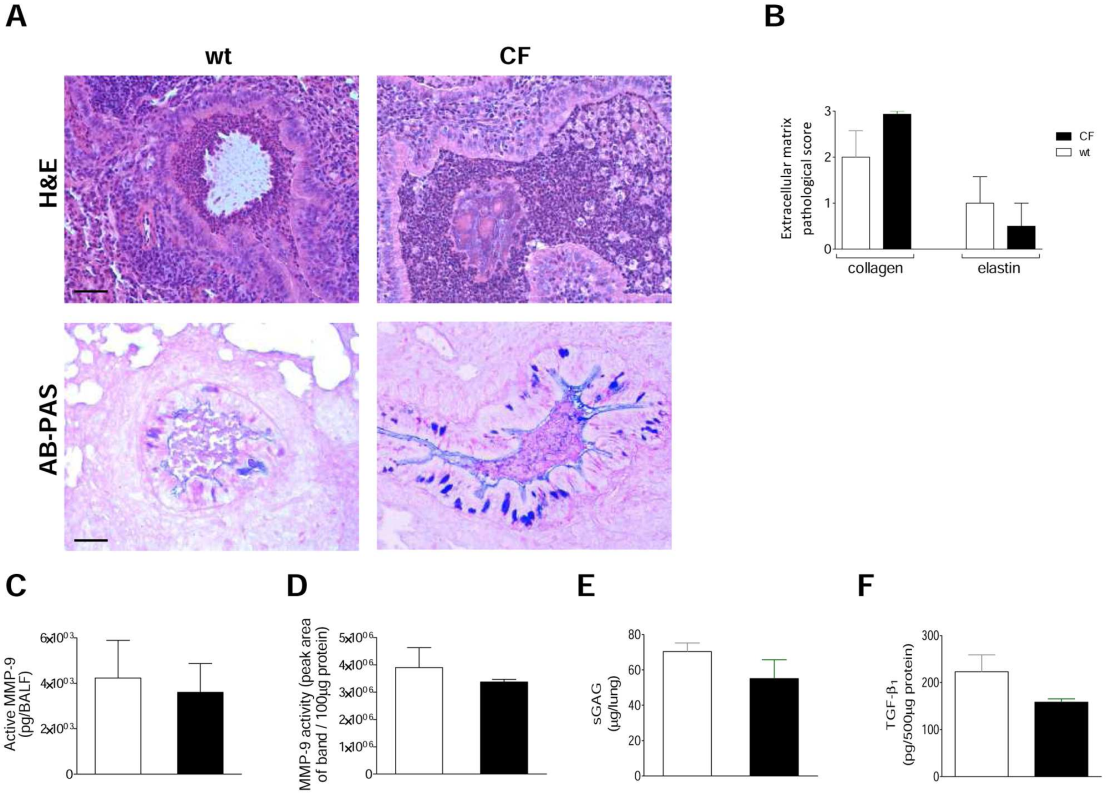

Fig S3. Tissue damage after *P. aeruginosa* lung chronic infection in CF and wt mice. Gut-corrected CFTR-deficient C57Bl/6 Cftr^{tm1UNC}TgN(FABPCFTR)#Jaw (CF) and congenic wt mice were infected with 2x10^{6} CFU/lung of *P. aeruginosa* AA43 isolate embedded in agar beads for 28 days. Sections of murine lungs were stained with H&E and AB/PAS (**A**) for mucopolysaccharides, according to the standard procedure. Scale bars: 50 µm. Scorings of collagen deposition and elastin degradation (**B**) were performed on slices stained with MTS and VEG, respectively. Levels of MMP-9 protein (**C**) in BALF by ELISA, MMP-9 activity (**D**) in lung homogenate by zymography, sGAG (**E**) in lung homogenate by a dye-binding colorimetric assay and TGF-β_1 (**F**) in lung homogenate by Bioplex were measured after 28 days of chronic lung infection with the *P. aeruginosa* CF-adapted isolate AA43. Values represent the mean ± SEM. The data are pooled from two independent experiments (n=3-11).

Table S1

Gene name	Gene symbol	Alternative common names	Macroarray results (respective to AA2)		Validation results Folds of induction [Mean ± SEM (N)]	
			AA43 vs AA2	AA44 vs AA2	AA43 vs AA2	AA44 vs AA2
Gro-b (Growth-regulated protein beta)	CXCL2	MIP-2a	down	down	0,53 ± 0.071 (N=5)	0.63 ± 0.085 (N=5)
Gro-g	CXCL3	MIP-2b	down	down	1.05 ± 0.213 (N=3)	0.95 ± 0.26 (N=3)
IL-8 (interleukin-8)	CXCL8		down	down	0.59 ± 0.041 (N=4)	0.54 ± 0.057 (N=4)
IP-10 (Interferon gamma-induced protein 10)	CXCL10	small-inducible cytokine B10	down	down	0.56 ± 0.111 (N=6)	0.67 ± 0.085 (N=6)
Angie	CXCL13	BLC (B lymphocyte chemoattractant)	up	up	nd	nd
C-C receptor 6	CCR6	cluster of differentiation (CD) 196	down	down	nd	nd
C-C receptor 7	CCR7	CD197	down	down	nd	nd
C-C receptor 9	CCR9	CDw199	down	down	nd	nd
CXC receptor 6	CXCR6	CD186	up	up	nd	nd

Intercellular adhesion molecule 1	ICAM-1	CD54	down	down	0.59 ± 0.117 (N=3)	0.51 ± 0.123 (N=3)
Vascular cell adhesion molecule 1	VCAM-1	CD 106	down	down	0.35 ± 0.072 (N=4)	0.61 ± 0.059 (N=4)
Adiponectin	ADIPOQ	GBP-28, apM1, Acrp30	down	down	nd	nd
Tumor Necrosis Factor alpha	TNF-a	cachexin	down	down	0.61 ± 0.047 (N=4)	0.58 ± 0.037 (N=4)
Toll-like receptor 2	TLR-2	CD282	down	down	0.96 ± 0.15 (N=3)	0.99 ± 0.057 (N=3)
Toll-like receptor 5	TLR-5		up	up	1.12 ± 0.12 (N=3)	1.24 ± 0.107 (N=3)
Human Defensin b1	HDB-1	DEFB1	up	up	nd	nd
Dual oxidase 2	FPR2	FPRL1	down	down	nd	nd
Statherin	STATH		down	down	nd	nd
Elastase 2	ELA2		down	down	0.89 ± 0.217 (N=3)	0.95 ± 0.34 (N=3)
Matrix metalloprotease 9	MMP-9	gelatinase B	up	up	0.97 ± 0.136 (N=4)	1.06 ± 0.101 (N=4)

nd: not determined

SUPPLEMENTARY METHODS

Infection of cell lines. IB3-1 and C38 cells were infected with *P. aeruginosa* isolates at a multiplicity of infection (MOI) of 0.1 for 4 h for RNA extraction and at a MOI of 1 for 2 h for analysis of IL-8 levels as previously described [4]. THP-1 cells were seeded and differentiated to macrophage-like cells as described previously [5], then infected for 4 h with *P. aeruginosa* isolates at a MOI of 1. Growth media were collected at the end of incubation, centrifuged and stored at –80°C for analysis of MMP-9.

Macroarray analysis and validation

Total RNA was extracted from lysed cells with the Total RNA Isolation kit (Roche), converted to cDNA with High Capacity cDNA Archive Kit (Applied Biosystems) and random primers.

In this study, the expression of 92 target genes, that are crucial in the first line of response against pathogens and critical in chronic inflammatory diseases of the airways, were obtained using TaqMan Low Density Array (TLDA) platform (Applied Biosystems, Foster City, CA), as previously described (1). To validate the data, separate quantitative Real-Time Polymerase Chain Reaction (qRT-PCR) experiments were performed as previously described (1-2). IL-8 and MMP-9 protein releases were measured with ELISA assays, according to the manufacturer's protocols (R&D).

Histological examination

Murine and human lungs were removed, fixed in formalin, and embedded in paraffin. Consecutive sections from the middle of the five lung lobes were used for histological, immunohistochemical, and immunofluorescence examination in each

mouse. Indirect immunofluorescence was performed using a polyclonal rabbit anti-*P. aeruginosa* Ab (kindly provided by G.B. Pier, Harvard Medical School, Boston, MA). The secondary Ab was Texas Red-labeled goat anti-rabbit Ig G (Molecular Probes). The slides were examined using an Axioplan fluorescence microscope (Zeiss), and images were taken with a KS 300 imaging system (Kontron). Sections for histological analysis were stained by H&E, Alcian Blue-Periodic Acid Schiff (AB-PAS), Masson's trichrome (MTS) and Verhoeff's elastic (VEG) staining and were examined blindly and scored by a pathologist, as describe below.

Bronchial epithelial degeneration was performed on lung sections stained with Haematoxylin-Eosin as previously described [6].

Histological score analysis of murine lungs was performed to grade the amount of innate immune cells infiltration and BALT activation. Histological examination primarily included the assessment of cellular infiltrates and aggregation by scoring the number of immune cells (mononuclear cells, such as macrophages, lymphocytes, plasma cells, and neutrophils) at a magnification of ×400. The number of inflammatory cells was evaluated by using a visual analogue scale modified for murine pulmonary specimens, as described previously [7], and results are reported as the mean for the entire specimen. When considerable heterogeneity of infiltration was evident in the same specimen, the mean for several areas was determined and the specimen was scored accordingly. Neutrophils and macrophages were classified as absent (score of 0) when there were no or fewer than 19 cells per high-power field (HPF) (at a magnification of ×400), mild (score of 1) for 20 to 49 cells per HPF, moderate (score of 2) for 50 to 99 cells per HPF, marked or severe (score of 3) for 100 to 200 cells or more per HPF. Histological criteria for normal pulmonary characteristics included detection of no or only a few mononuclear cells per HPF and

no or only a few scattered neutrophils in bronchioli and alveoli without tissue changes (no interstitial thickening or aggregates of lymphocytic infiltrates, and airways free from exudate). The number of inflammatory cells and of lymphoid aggregates, assessed at ×400 and ×100 magnification respectively, was scored and customized as described by Martino et al [8].

Histologic lesions of the respiratory bronchial epithelium were evaluated and scored for the presence or absence of ciliated columnar cells, goblet cells, edema, hyperplasia or metaplasia of epithelial cells, and lymphocytes infiltration (see also the scoring system to evaluate adaptive immunity), based on the previously described scoring system [9-11]: presence of ciliated columnar epithelium, normal goblet cells, and no lymphocyte infiltration in respiratory epithelium (score 1); presence of focal lesions, some degenerative or necrotic epithelial cells, small focal areas lacking cilia, no lymphocyte infiltration in respiratory epithelium (score 2); multifocal areas lacking cilia accompanied by edema, degenerative and hyperplastic changes in epithelial cells, locally disrupted epithelial layer, infiltration of lymphocytes (score 3); diffuse or severe lesions showing replacement of normal ciliated columnar epithelial lining by the squamous to cuboidal epithelium without cilia, disrupted epithelial layer in many places, depletion of goblet cells, and infiltration of lymphocytes into respiratory epithelium (score 4). The sums of scores of bronchial lesions for mouse in one group were summed, and was used for statistical comparison of the severity lesions between the groups. Finally, concerning histological scores indicating an adaptive immune response, the presence of lymphoid nodules consisting of 5 to 7 lymphocyte-like cells (germinal centers) within areas of secondary bronchi and blood vessels, the intensity of dispersed lymphocyte infiltration in the interalveolar septa and interparabronchial septum and around the secondary bronchi as well as around the blood vessels was

evaluated and recorded. The score was calculated as follow: no lymphocyte accumulation in interalveolar and interparabronchial septa, and very few germinal centers around secondary bronchi and blood vessels (score 0); accumulation of a few dispersed lymphocytes without the increase of the number of germinal centers (score 1); moderate accumulation of lymphocytes in interalveolar and interparabronchial septa, increased size of germinal centers (group of 10–13 cells) around blood vessels (score 2); thickened interatrial septa in a large area of the lung, interparabronchial septa infiltrated with histiocytes and lymphocytes, and increased number of germinal centers (score 3). The sum of scores of the mice in one group was used for statistical comparison of the severity of bronchial lesions and mononuclear cells system activation between the groups.

For BALT activation, the score was assessed at ×100, by calculating the total areas of BALT follicles evaluated inside each lung section, and subdividing the mean as follow: mean area of BALT follicle extension none (score 0); up to 0.008 square mm (score 1); up to 0.042 square mm (score 2); up to 0.4 square mm (score 3).

For evaluation the degree of fibrosis in lungs, Masson’s trichrome-stained (MTS) sections were assessed as follow: the areas of lung fibrosis were represented as blue-stained areas, and the parenchyma as red-stained regions by MTS. The total area of the section was the sum of the area of all microscopic fields, including parenchyma and fibrosis. Elements of the pleura were excluded from the computations. Then the percentage of bluish-green stain, representing the area of fibrosis for each lung section specimen, was calculated using the Leica Qwin 500 Image Analyzer (Leica, Cambridge, England), to capture the widest area of tissue. Images of trichrome stained sections were captured using a x 5 HPFs and the area of the section was the sum of the area of all microscopic fields, including parenchyma and fibrosis. The

scoring system was characterized as follow: normal lung (score 0); lungs showing a minimum percentage of fibrosis (percentage area of fibrosis ≥1.709; ≤ 6.899; mean area 4,304) (score 1); moderate (≥6.899; ≤ 10.798; mean percentage area 8.848) (score 2); severe (corresponding to an area ≥10.798; until 32.498 and over; mean percentage area 19,215) (score 3).

Verhoeff's elastic stains (VES) was used for assessing and quantifying the elastin architecture in lung interstitial and peribrochial areas. The degree of fragmentation and the amount of the elastic fibers were examined by the pathologist, blinded to the treatment group, who scored each slide using an arbitrary combined scoring system that counted the number of “islands of damage” within a lung cross-section from each mouse. An island of damage was defined as an isolated area of lung’s interstitium or peribronchus, where two adjacent elastic fibers were fragmented with interposed excessive connective tissue matrix, evaluated randomly on the cross-section at x 400. Three evaluations were performed per lung. The method was extrapolated from McLoughlin *et al.* [12].

Evaluation of markers associated to tissue damage. Murine active MMP-9 and sGAG were measured respectively by ELISA (R&D) and by sGAG assay (Kamiya Biomedical Company), according to the manufacturers’ instructions. Collagen was quantified by Sircol Collagen Assay (Biocolor Life Science) according to manufacturer’s instructions. Gelatinase zymography was performed to evaluate the activity of MMP-9 (gelatinase B) in murine BALF and lung homogenates [13]. Protein concentration was determined; 100 µg of lung homogenates were analyzed. Supernatants (SN) from macrophages-like cells THP-1 were used as positive control. Samples and molecular weight (BlueStar Protein Marker; Life Science) were

electrophoresed on precast polyacrilamide gel with gelatine (10%). After electrophoresis the gels were incubated in Renaturing Solution (2,5% Triton X-100) for 30 min at room temperature. The gels were equilibrated in Developing Solution (50 mM Tris, 200mM NaCl, 5mM CaCl2 and 0,02% Brij-35, pH 7.5) for other 30 minutes and then incubated at 37°C in the same buffer for 24 hrs. They were stained for 40 minutes with Staining Solution (0,5 % Comassie Brilliant Blue in 45% methanol and 10% acetic acid) and then destained for 2 hrs with Destaining Solution (45% methanol and 10% acid acetic). Zones of murine and human MMP-9 proteolysis appeared as clear bands against a blue background at approximately 92 kDa and 85 kDa respectively. Band intensities were measured by ImageJ. Data obtained were normalized to the value of the positive control.

Cytokines/chemokines quantification

Bio-Plex® multiplex system was used for the quantification of cytokines/chemokines and growth factors, according to the manufacturer's instruction. In details, Multiplex immunoassays (Bio-Rad) based on Luminex technology were used for the quantification of cytokines, chemokines and growth factors in murine samples, according to the manufacturer's instruction. A mouse Bio-Plex custom mix was used to analyze MIP-2, KC, MIP-1α, IL-6, MCP-1 and TNF-α in murine lung homogenates. Results were expressed as pg/500ug protein of lung homogenate.

The three isoforms of TGF-β (TGF-β_1, TGF-β_2 and TGF-β_3) were analyzed with Bio-Plex Pro™ TGF-β 3-plex panel in murine BAL fluid and lung homogenates.

Data were measured on Bio-Plex 200 System and calculated using Bio-Plex Manager 6.0 and 6.1 software. Levels were expressed as pg/total BALF and pg/500ug protein of lung homogenate for murine samples.

SUPPLEMENTARY REFERENCES

1 Bragonzi, A. *et al.* Sequence diversity of the mucABD locus in Pseudomonas aeruginosa isolates from patients with cystic fibrosis. *Microbiology* **152**, 3261-3269, doi: 10.1099/mic.0.29175-0 (2006).

2 Bragonzi, A. *et al.* Pseudomonas aeruginosa microevolution during cystic fibrosis lung infection establishes clones with adapted virulence. *Am J Respir Crit Care Med* **180**, 138-145, doi:10.1164/rccm.200812-1943OC (2009).

3 Cigana, C. *et al.* Pseudomonas aeruginosa exploits lipid A and muropeptides modification as a strategy to lower innate immunity during cystic fibrosis lung infection. *PLoS One* **4**, e8439, doi:10.1371/journal.pone.0008439 (2009).

4 Bianconi, I. *et al.* Positive signature-tagged mutagenesis in Pseudomonas aeruginosa: tracking patho-adaptive mutations promoting airways chronic infection. *PLoS Pathog* **7**, e1001270, doi:10.1371/journal.ppat.1001270 (2011).

5 Verma, N., Chaudhury, I., Kumar, D. & Das, R. H. Silencing of TNF-alpha receptors coordinately suppresses TNF-alpha expression through NF-kappaB activation blockade in THP-1 macrophage. *FEBS Lett* **583**, 2968-2974, doi:10.1016/j.febslet.2009.08.007 (2009).

6 Monticelli, L. A. *et al.* Innate lymphoid cells promote lung-tissue homeostasis after infection with influenza virus. *Nat Immunol* **12**, 1045-1054, doi:10.1031/ni.2131 (2011).

7 Cersini, A., Martino MC, Martini I, Rossi G, Bernardini ML. Analysis of virulence and inflammatory potential of Shigella flexneri purine biosynthesis mutants. *Infect Immun.* **71**, 7002-7013 (2003).

8 Martino, M., Rossi G, Martini I, Tattoli I, Chiavolini D, Phalipon A, Sansonetti PJ, Bernardini ML. Mucosal lymphoid infiltrate dominates colonic pathological changes in murine experimental shigellosis. *J Infect Dis.* **192**, 136-148 (2005).

9 Dykstra, M., Levisohn S, Fletcher OJ, Kleven SH. Evaluation of cytopathologic changes induced in chicken tracheal epithelium by Mycoplasma gallisepticum in vivo and in vitro. *Am J Vet Res.* **46**, 116-122 (1985).

10 Nili, H., Asasi K. Avian influenza outbreak in Iran. *Avian Dis.* **47**, 828–831 (2003).

11 Noormohammadi, A., Jones JF, Harrigan KE, Whithear KG. Evaluation of the non-temperature-sensitive field clonal isolates of the Mycoplasma synoviae vaccine strain MS-H. *Avian Dis.* **47**, 355-360 (2003).

12 McLoughlin, D. *et al.* Pravastatin reduces Marfan aortic dilation. *Circulation* **124**, S168-173, doi:10.1161/CIRCULATIONAHA.110.012187 (2011).

13 Bergin, D. A. *et al.* Airway inflammatory markers in individuals with cystic fibrosis and non-cystic fibrosis bronchiectasis. *J Inflamm Res* **6**, 1-11, doi:10.2147/JIR.S40081 (2013).
